# Does Topical Capsaicin Affect the Central Nervous System in Neuropathic Pain? A Narrative Review

**DOI:** 10.3390/ph17070842

**Published:** 2024-06-27

**Authors:** Kareem Alalami, Jenna Goff, Hannah Grimson, Oliver Martin, Eloise McDonald, Thonima Mirza, Dhruvi Mistry, Adanma Ofodile, Sara Raja, Tooba Shaker, Danah Sleibi, Patrice Forget

**Affiliations:** 1 School of Medicine, Medical Sciences and Nutrition, University of Aberdeen, Polwarth Building, Foresterhill Health Campus, Aberdeen AB24 3FX, UK; k.alalami.22@abdn.ac.uk (K.A.); d.sleibi.21@abdn.ac.uk (D.S.); 2 Epidemiology Group, Institute of Applied Health Sciences, School of Medicine, Medical Sciences and Nutrition, University of Aberdeen, Polwarth Building, Foresterhill Health Campus, Aberdeen AB25 2ZN, UK; 3 Department of Anaesthesia, NHS Grampian, Aberdeen AB25 2ZN, UK; 4 Pain and Opioids after Surgery (PANDOS) Research Groups, European Society of Anaesthesiology and Intensive Care, 1000 Brussels, Belgium; 5 Anesthesia Critical Care, Emergency and Pain Medicine Division, 30900 Nîmes University Hospital, IMAGINE UR UM 103, Montpellier University, 30900 Nîmes, France

**Keywords:** topical capsaicin, chronic pain (neuropathic), TRPV1, CNS (Central Nervous System), PNS (Peripheral Nervous System)

## Abstract

Research has been conducted investigating the neuronal pathways responsible for the generation of chronic neuropathic pain, including the components of it in conditions such as chronic post-surgical pain, phantom limb pain, and cluster headaches. Forming part of the management of such conditions, capsaicin as a molecule has proven effective. This review has investigated the central nervous system modifications exhibited in such conditions and the pharmacological mechanisms of capsaicin relevant to this. The current paradigm for explaining topical capsaicin-induced analgesia is that TRPV1-mediated calcium ion influx induces calpain, in turn causing axonal ablation and functional defunctionalisation in the PNS (Peripheral Nervous System). Demonstrated through the analysis of existing data, this review demonstrates the changes seen in the CNS (Central Nervous System) in chronic neuropathic pain, as well as some of the evidence for capsaicin modulation on the CNS. Further supporting this, the specific molecular mechanisms of capsaicin-induced analgesia will also be explored, including the action of TRPV1, as well as discussing the further need for clinical research into this area of uncertainty due to the limited specific data with suitable parameters. Further research this review identified as potentially useful in this field included fMRI (functional Magnetic Resonance Imaging) studies, though more specific observational studies of patients who have already been administered capsaicin as a current treatment may prove helpful in studying the modification of the CNS in the long term.

## 1. Introduction

Chronic pain affects more than 30% of people worldwide [1]. In the United Kingdom, between one-half and one-third of the population experience chronic pain, which correlates to nearly 28 million people [2]. Chronic pain is defined as pain that lasts for three or more months [3]. Chronic pain can be mechanistically categorised into three groups: neuropathic pain (pain caused by disorder or injury to the somatosensory system cortex), nociceptive pain (pain due to tissue injury or disorder), and mixed pain (copresence of neuropathic and nociceptive pain) [4]. Central sensitisation has been shown to play a role in pain, a process by which there is an alteration to the structure and function of the central nervous system (CNS), which leads to the brain and spinal cord becoming hypersensitive to pain [5]. Capsaicin may be a promising drug for use in the management of neuropathic chronic pain [6].

Capsaicin (8-methyl-N-Vanillyl-6-nonenamide) is an active compound found in red chilli pepper, belonging to a group of compounds called vanilloids. Capsaicin has been found to provide effective, long-lasting, and faster onset analgesia with few systemic side effects. Some of the common side effects predominantly have dermatological manifestations, including papules, pruritus, and erythema. Capsaicin is administered in various modalities including sprays, ointments, lotions, and patches. Historically, topical capsaicin was given in low concentrations (<1%), which needed regular and ongoing administration. However, studies have demonstrated a higher efficacy of capsaicin’s analgesic effects when used in high concentration (8%) [7]. The mechanism of action of capsaicin is that it acts on the transient receptor potential vanilloid 1 (TRPV1) channels, which are found in many cell types. Examples of these include immune, neuronal, epithelial, and muscular cells. This could indicate that capsaicin affects various parts of the body, therefore suggesting it could be used to manage an extensive range of conditions [8]. This paper will aim to provide understanding on how topical capsaicin could be used to manage chronic neuropathic pain by exploring its mechanism of action on the central nervous system. 

## 2. Methods

This study is a narrative and qualitative review of the evidence surrounding capsaicin’s effect on the CNS regarding the treatment of chronic neuropathic pain. We searched the existing literature focussing on topical capsaicin and its analgesic properties, primarily using PubMed/MEDLINE due to the high availability of published data on the topic; we also recognise this as a limitation due to the relatively low volume of published data on the topic as a whole. Key words used in the search criteria included “Capsaicin”, “Central Nervous System”, and “Neuropathic Pain”, which aided in identifying suitable data. We identified articles describing the CNS modulation seen in neuropathic pain as well as looking at this after topical capsaicin use, in humans. References were screened further by selecting the papers that we believed provide a fair and accurate depiction of the capacity in which capsaicin is currently used and that we felt suggested the potential capabilities of capsaicin that were yet to be fully comprehended without being too speculative. We also considered both direct and indirect evidence, demonstrating a mix of research styles ranging from trial data to observational. We consensually selected 58 papers that we believe represented the highest quality studies that have been conducted on capsaicin relevant to this field, along with articles related to the pathophysiology of certain worked examples of chronic neuropathic pain. The papers selected are a mix of systematic reviews, narrative reviews, clinical trials, and other studies both in vivo and in vitro. Evidence was analysed from a variety of different administration methods of the drug to also observe whether administration route had any effect on how the capsaicin provided pain relief. Research regarding the specific pathophysiology of chronic neuropathic pain has also been included in the scope of this review in order to give a basic context of current capsaicin use, and how this proves effective in conditions it is currently used to treat.

### 2.1. Pain

#### 2.1.1. Physiology of Pain

**Figure 1:**
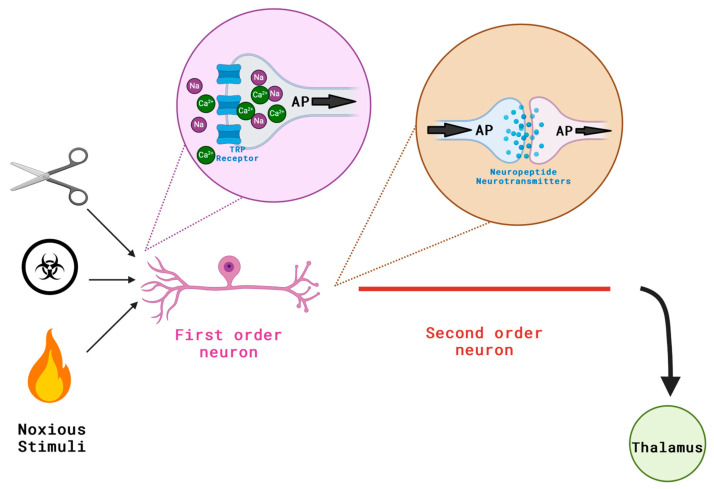
Illustration of the pain pathway. In pain pathways, noxious stimuli (mechanical, chemical and heat) activate the TRP receptors on the first order sensory neurons; this causes an influx of sodium and calcium ions, leading to membrane depolarisation and thus generating an action potential (the frequency of which codes for the intensity of the stimulus). This is also potentiated by an inflammatory response. The central terminals of the first order neurons then release neuropeptide neurotransmitters (e.g., glutamate, substance P) which bind to receptors on the second order neurons and allow for the continuation of the action potential and in turn the pain pathway. AP = action potential. Created with BioRender.com (license BX26ZL4PCY).

**Figure 2:**
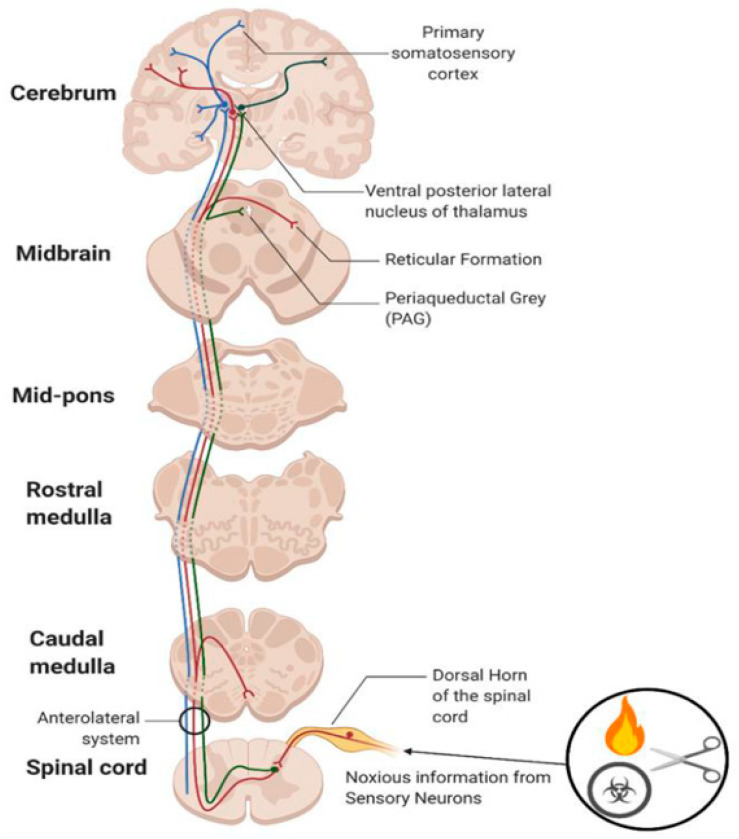
Illustration of the spinothalamic tract. The pathway that senses noxious stimuli and decussates at the vertebral column, before reaching the brain which is where the relevance comes into play for analgesics. (Adapted from [10]).

**Figure 3:**
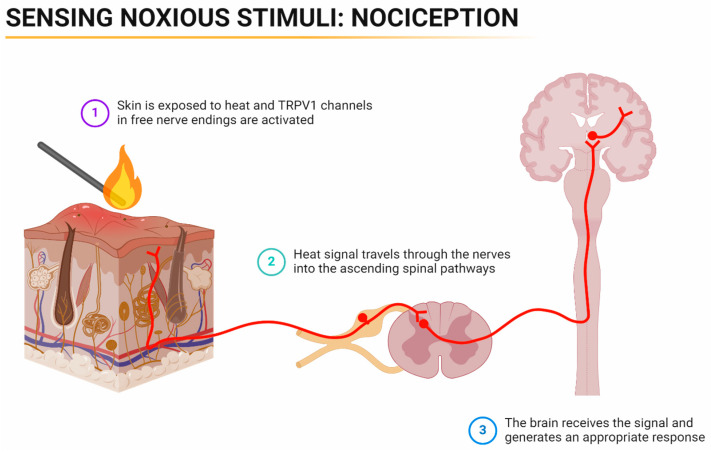
Illustrating the ascending nociceptive pathway. TRPV1 = transient receptor potential vanilloid 1. Created with BioRender.com (license QZ26ZLBGSQ).

Pain is an intricate, unpleasant blend of sensory and emotional experiences that are linked to actual or potential tissue damage. It happens when specific sensory neurons are activated by chemical, mechanical, or thermal stimuli [9] (Figure 1). The noxious stimuli activate sensory neurons that trigger a series of events in the nervous system. These neurons send signals to the spinal cord and brain, which process and interpret them as pain signals (Figure 2). Nociception refers to the perception of potentially harmful stimuli by the nervous system, which helps to facilitate future responses to the stimuli [10] (Figure 3). Nociceptors are types of sensory neurons that convert these stimuli into electrical signals. These are classified into two types: Aδ and C fibres. Aδ fibres are myelinated, facilitating the swift conduction of well-localised nerve signals [11]. C fibres are unmyelinated, have a smaller diameter and have a slower conduction, causing a long-lasting burning sensation that is poorly localised [11].

#### 2.1.2. Chronic Pain

The World Health Organization (WHO) [12] updated the International Classification of Diseases (ICD-11) to include a novel way of categorising chronic pain. Pain is divided into chronic primary pain and chronic secondary pain. Chronic primary pain persists over three months and is accompanied by significant mental and emotional distress, and rather than a symptom of a chronic condition this pain is a standalone health condition itself. Among chronic primary pain disorders are fibromyalgia, complex regional pain syndrome, chronic migraine, and irritable bowel syndrome. Conversely, chronic secondary pain initially appears as a symptom of another condition and is diagnosed when the pain itself becomes a problem. Even after successful treatment, pain may persist. As well as headaches or nerve damage, chronic secondary pain can result from cancer, surgery, and injury, as well as muscle, bone, and joint disease [12].

#### 2.1.3. Neuropathic Pain

**Figure 4:**
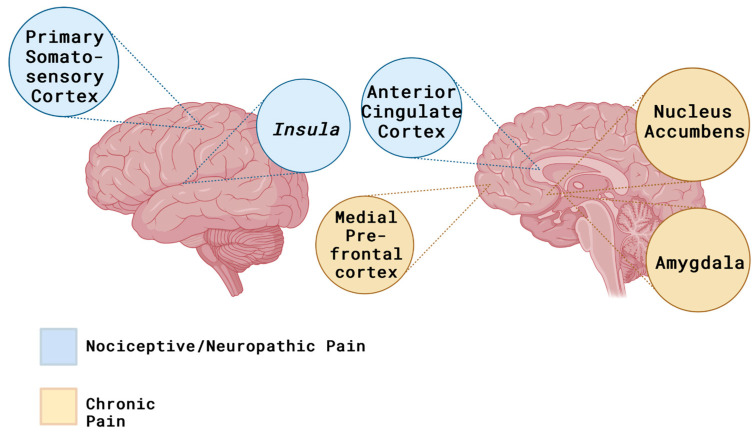
Areas of the brain stimulated by nociceptive/neuropathic pain (acute) and chronic pain. Due to the persistence involved in chronic pain, the brain undergoes changes in the functional connections that allow for the processing of pain. These changes show a divergence from acute sensory circuits to emotional circuits. Created with BioRender.com (licensePV26ZL42QV).

Neuropathic pain is caused by an injury or disease that affects the somatosensory nervous system [1]. Features that are apparent in many cases of neuropathic pain are continuous burning pain, paroxysmal pain, pain caused by a stimulus that would not normally cause pain, such as a light feather touch, and hyperalgesia to name a few. Common conditions where neuropathic pain can arise from include a stroke, spinal cord injury, brain trauma, and multiple sclerosis (MS). The mechanisms involved in the neuropathic pain pathway are quite diverse and travel from the periphery to the brain. The main areas of the brain involved are the primary somatosensory cortex, the anterior cingulate cortex, and the prefrontal cortex (Figure 4). These key areas are responsible for how and why pain is felt, and the intensity at which it is experienced [1]. Specifically, the prefrontal cortex is where painful or noxious stimuli are processed, in response creating sensory perception, cognition, and an emotion to produce the outcome. Neuropathic pain is caused by changes, specifically structural and functional, which increase the progression of the pain [1].

In cases where there is tissue damage, chemical mediators will create an area of primary hyperalgesia. This allows general localisation of the damage and the result of the peripheral sensitisation of the nociceptive pain fibres. If there is continued activation of the nociceptive fibres, this will also affect the 2nd order nociceptive neurons creating a secondary area of hyperalgesic sensation. This second area that has been stimulated has not sustained any damage, but it is experiencing an increase in sensitivity to the painful stimuli as a “knock-on” effect [13,14].

### 2.2. Capsaicin on Neuropathic Pain

**Figure 5:**
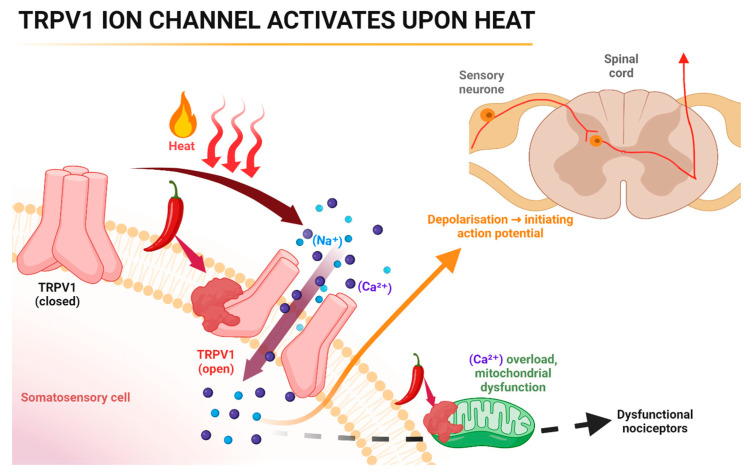
Mechanism of action of capsaicin in the periphery. Capsaicin activates TRPV1 which sensitises the area to heat, acidosis, and endogenous agonists. Symptoms include itching, stinging, burning, or heat. Repeated high concentration exposure defunctionalises cutaneous nociceptors, reducing spontaneous activity and responsiveness to stimuli. The figure represents capsaicin induced Ca^2+^ influx through TRPV1 channel triggers desensitisation and neurotoxicity but the downstream molecular mechanisms are still unclear. Mitochondrial swelling is an early sign of neurotoxicity. Ca^2+^ sequestration in mitochondria triggers apoptosis via caspase activation. Resiniferatoxin can provide permanent analgesia in cancer patients by ablating sensory neurons. TRPV1 activation by agonists is inhibited by its antagonists, leaving other pain sensors intact. TRPV1 = transient receptor potential vanilloid 1. Created with BioRender.com (license JP26ZL5U3X).

The capsaicin receptor, called transient receptor potential cation channel, subfamily V member 1 (TRPV1), is highly expressed specifically in the type C sensory neurons [11]. TRPV1 is a cation channel that allows for the permeation of Ca^2+^ ions and is primarily found in the spinal cord and peripheral terminals of non-myelinated primary afferent neurons [15]. TRPV1 receptors play a crucial role in acute heat nociception and inflammatory hyperalgesia in sensory neurons (Figure 5). 

Capsaicin affects the PNS by activating the receptor TRPV1 (a pain receptor) and creating a burning sensation as this receptor opens; however, it also has analgesic properties. The mechanism of action of this is thought to be through the desensitisation of TRPV1 within the periphery [16]. This prevents some of the excitatory currents of Na^+^ voltage-gated channels and thus decreases the reactivity of the sensory neurons in the periphery. After this, it blocks conductance, resulting in a reduced sensitivity in peripheral nociceptive endings. In some cases, it can even cause irreversible degenerative changes to nerves due to neurotoxicity [17]. TRPV1 action can also be seen in some of the neurons and glial cells. This results in there being some effect of capsaicin within the CNS [16,18,19].

In neuropathic pain, there is an upregulation in TRPV1, which increases the sensation of pain. The mechanism of action of capsaicin in neuropathic pain analgesia is that initially, it depolarises TRPV1 when it binds to it—this is the cause of the burning feeling. Continuous exposure to capsaicin or a higher concentration of it will eventually cause TRPV1 to be defunctionalised—this is what causes the analgesic effect [8,20,21,22].

Capsaicin is a selective compound that only stimulates a few nociceptive neurons. It activates TRPV1 and does not have the same affinity for the other receptors of the vanilloid receptors i.e., TRPV2-6 [20]. Currently, some studies show that capsaicin and its activity on TRPV1 can be used advantageously within pain pathways, such as the inflammatory pain pathway, by targeting some of the capsaicin-triggered TRPV1 receptors [20].

### 2.3. Neuromodulation in Capsaicin-Induced Analgesia

The long-lasting analgesic effect exerted by capsaicin is known to be mediated by axonal ablation and there are several mechanisms by which capsaicin can induce ablation of nociceptor nerve terminals. It was established in an in vitro study that capsaicin-induced ablation of axonal terminals is primarily achieved through TRPV1-mediated influx of calcium ions [7]. The key step in analgesia induced by capsaicin is the initial defunctionalisation and desensitisation of TRPV1, as this plays a key role in activating many downstream molecular pathways. 

Consistent with its role in the periphery, the TRPV1 receptor in the brain is important in pain transmission and modulation [23]. An in vivo study observed the generation of anti-nociceptive effects when microinjections of capsaicin were administered in the periaqueductal region of the brain, which is rich in TRPV1 receptors [24]. In previous studies, capsaicin has been shown to activate PKA and PKC, phosphorylating the NMDA receptor [1]. It has been shown that persistent activation of the NMDA receptors causes hyperresponsiveness in the spinal cord neurons, owing to central sensitisation, forming part of the pathophysiology of chronic pain [25].

Mitogen-activated protein kinases (MAPKs) are also activated in glial cells by nerve or spinal cord injury. Their activation can induce peripheral sensitisation in the nociceptive primary sensory neurons and central sensitisation in the spinal cord dorsal horn neurons [26]. Moreover, an increase in MAPK phosphorylation is notable after capsaicin administration showing a potential link in capsaicin’s ability to modulate this pathway [24].

Another similar finding revealed that capsaicin administration, in combination with increased cAMP concentration, in the trigeminal ganglion, results in hyperalgesia and allodynia. An in vitro study found that an excitatory response is generated upon first application of capsaicin to sensory afferent nerves; however, after capsaicin receptor desensitisation has taken place, the amplitude of the excitatory response gradually begins to drop. This decline is presumably due to inhibition of APs (Action Potentials) via blockage of voltage-gated sodium channels. This indicates selective modulation of voltage-gated channels and action potentials (AP) by capsaicin, which may occur due to the presence of other secondary messenger pathways, leading to sensitisation or desensitisation [21]

It is proposed that in the CNS, capsaicin-induced TRPV1 activation in the microglia elicits an increase in intracellular calcium concentration and stimulates cytochrome c release from the mitochondria, resulting in microglial death [21]. However, different studies have proposed different pathways that are yet to be explored in more detail surrounding this exact mechanism. Therefore, the findings related to the capsaicin-induced effect in the CNS are still paradoxical.

### 2.4. Conditions and Clinical Applications

#### 2.4.1. Capsaicin Use in Chronic Post-Surgical Pain (CPSP)

Chronic pain arising from surgical procedures in the post-operative period is a major side effect, estimated to affect anywhere from 10–30% of surgical patients after 1 year postoperatively [27], with an incidence of moderate to severe CPSP at 12 months of 11.8% [28]. Chronic post-surgical pain is defined as the following [29]: Pain that is experienced for at least 3 months post-operatively, that was non-existent preoperatively, or that had differed in intensity or characteristics from the pain before surgery. The pain should be localised to the surgical site or a referred area, with the exclusion of other possible causes of the pain (e.g., cancer recurrence, infection). Neuropathic pain itself may not simply induce neuroplastic changes in the peripheral nervous system (PNS) but also in the central nervous system (CNS), specifically in the somatosensory cortex (S1) [30]. This evidence, backed by fMRI data, has shown that not only is it a by-product of neuropathic pain, but may contribute to it as a cause further propagating the pain and sensitisation pathways with time.

Looking first at in vivo models of capsaicin for CPSP, there is data supporting significantly reduced allodynia and hyperalgesia in rats exposed to mechanical pain when compared to rats that did not receive the treatment of high-concentration intradermal capsaicin. This was dependent on the timing before exposure to stimuli as this varied (e.g., 6 days versus 24 h) [31]. In humans, a systematic review of previous studies chose to reconsider evidence from double-blind randomised control trials (RCTs) looking at several causes of neuropathic pain (such as post-herpetic neuralgia and HIV-neuralgia). They concluded that while the trial data were of poor sufficiency, there is similar efficacy and effects when compared to other therapies for chronic pain [32], potentially opening the door to applying this in neuropathic CPSP. In the ASCEND study, this further proved the patch form of capsaicin is an effective, long-lasting, and well-tolerated analgesic option for neuropathic pain [33,34,35].

#### 2.4.2. Capsaicin Use in Cluster Headaches

Cluster headaches are a trigeminal autonomic cephalgia affecting seven million people around the world [36]. It causes intense, severe attacks of pain ranging from short periods to long periods [37]. This is quite consequential. 

Patients suffering from this agonising pain tend to have symptoms of blocked nose, runny nose, nausea, watery red eyes on the affected side, and burning piercing pain unilaterally on the face [37].

Research indicates that the central nervous system route is responsible for the cognitive processing malfunctions leading to the development of cluster headaches [36]. In another study, it was suggested that during these painful attacks, there is more than one chemical mediator responsible: Neurokinin A, calcitonin gene-related peptide, and substance P were shown to be elevated during acute attacks [38]. A similar study showed that due to an elevation of these chemicals, there is an activation of trigeminal vascular afferent nerves that later causes vasodilation, contributing to the propagatory effect cluster headache can possess [36]. From these data, we know that on a molecular level that there is dysregulation of certain chemicals causing cluster headaches to occur. 

The network handling nociceptive afferents from A-delta nociceptors is implicated in cluster headaches, which have an impact on the central nervous system [39]. This sensitisation reduces the threshold for pain perception and increases responsiveness to nociceptor activation [39]. In real terms, this means that patients will feel pain faster as their A-delta nociceptors are increasingly sensitive with time. It was shown in a study that topical capsaicin may reduce the number of substance P nerve terminals, adding to the theory that it may desensitise the sensory neurons contributing to cluster headaches [40]. 

With the current medications available for cluster headaches, like triptans, there are also significant side effects involved. Triptans can cause dizziness, coronary vasoconstriction, nausea, paraesthesia, tingling, neck pain, flushing, and chest tightness [41,42]. With capsaicin posing a well hypothesised argument, a role for it in treating cluster headache may prove a better alternative once both true and relative efficacy have been determined. 

#### 2.4.3. Phantom Limb Pain

Phantom limb pain (PLP) is clinically described as experiencing discomfort or pain in a missing limb [43]. It is still unclear what the underlying pathophysiology of PLP is. It was initially thought that the main mechanisms were only at the periphery; however, recent developments in laboratory methods and imaging (MRI and PET scans) have demonstrated indications of central nervous system (CNS) involvement [43].

During amputation, trauma occurs to the nerves and proximal tissues. This leads to disruption of the efferent and afferent signals in the amputated limb and formation of neuromas, which increases voltage-gated sodium channel expression, subsequently increasing excitability [43]. The CNS receives sensory afferents from the peripheral nervous system (PNS) through the dorsal root ganglia (DRG), meaning an increase in excitability causes continuous ectopic firing from the DRG. The DRG’s abnormal firing activates the nociceptive pathway, causing an unusual and spontaneous sensation of pain, even in the lack of any actual external stimulus. The net effect of this is creating a physiological response of central sensitisation in the spinal cord [44]. The dorsal horn’s NMDA receptors are crucial in the initiation of central sensitisation. Elevated stimulation of these receptors, coupled with spinal cord neuroplastic changes, is suggested as a mechanism contributing to neuropathic alterations significant in chronic and PLP post-amputation [45].

Multiple neurophysiological and neuroimaging investigations have indicated that cortical reorganisation (remapping) is linked to limb amputation. A notable outcome of this reorganisation is the development of PLP [46]. Cortical reorganisation is primarily characterised by neighbouring neurons invading the deafferented cortex, leading to the replacement of the lost sensorimotor modality with another. For example, studies that looked at motor cortices of amputees of the upper limb with PLP using functional magnetic resonance imaging (fMRI) have shown that areas originally occupied by the hand may be invaded by cortical areas of the lip [46], and a simple task such as lip-pursing activates the motor and sensory lip/face area but also extended to the motor hand area in the contralateral hemisphere to the amputation [47]. 

Several therapeutic options have been used for the treatment of PLP, including nortriptyline, pregabalin, opiates, and ketamine, which can effectively treat PLP by blocking receptors linked to central sensitisation and enhancing nerve inhibition to reduce pathologic stimulation [48]. However, these can cause adverse effects, for example drowsiness as well as more severe side effects such as dependence, and result in poor compliance as a result. According to a study, four weeks of applying an 8% capsaicin patch for 60 min to the amputation stump successfully lowered pain in both the stump and the phantom limb, with no systemic side effects or adverse events documented [49]. In the same study, patients were assessed with fMRI scans. The distribution of lip-to-hand representation, triggered during the lip-pursing task in the cortex opposite to the amputation stump, decreased in amputees who reported relief from PLP. Functional MRI analysis offered objective proof of the restored brain maps, suggesting that the localised effects of the capsaicin 8% patch can reduce PLP (“central” pain) and that peripheral inputs can have an impact on PLP, even in the CNS [49].

### 2.5. Summary of Formulations of Capsaicin 

Capsaicin is available in the following forms in the United Kingdom: cutaneous cream 0.025%, cutaneous cream 0.075%, and cutaneous patch 179 mg (8%). These are prescription only, with a drug tariff price of £210, and NHS indicative price of £210 [34]. In comparison, an analgesic that may be similarly prescribed, for example lidocaine hydrochloride patch 700 mg, the drug tariff price is £72.40, with an NHS indicative price of either £61.54 or £72.40 (depending on brand) [35]. Considering the longevity of treatment as well as efficacy, this also may play into a cost effectiveness argument in health systems with limited resources. 

## 3. Discussion

While the analgesic effects of capsaicin on the PNS are evident, its effect on the CNS has mixed evidence. Multiple studies were found supporting the hypothesis that pain sensation in the CNS can be modulated by capsaicin, both positively and negatively (by potentially causing sustained hyperalgesia). There is also an argument to be made about whether the CNS remodelling seen in chronic neuropathic pain is a direct effect of the capsaicin treatment, or the remodelling changes of the chronic neuropathic pain itself demonstrated, for example, in chronic post-surgical pain [30].

Briefly recapping the current proposed mechanism of action, topical capsaicin stimulates the TRPV1 receptor on neurons, leading to the opening of ion channels and causing positive cation influx including Na^+^ and Ca^+^. This leads to depolarisation of the cell and the initial hypersensitisation of the neuron, later defunctionalising the neuron via its neuro-ablative effects on the nerve endings. Four potential CNS mechanisms other than the TRPV1 action were found with a possibility of modulating pain due to capsaicin. One such theory can be explained by the suggestion of capsaicin causing central hypersensitisation. The activation of MAPK pathways causes the release of neurotransmitters CGRP (calcitonin gene-related peptide), substance P, and neurotrophin BDNF (brain-derived neurotrophic factor), which in turn activate NMDA receptors [26,50]. However, this combined central and peripheral sensitisation is theorised to lead to persistent allodynia and hyperalgesia even when inflammation subsides post-capsaicin application [51], making capsaicin application in chronic neuropathic pain controversial if it was to be applied in such a manner. Further to this, there is not enough evidence that a period of central hypersensitivity can lead to central desensitisation or will taper as seen peripherally with capsaicin. Further research on the molecular pathways involved in acute and chronic central hypersensitisation is thus required to determine whether capsaicin has a net positive or negative effect, both in treating chronic neuropathic pain, but also conveying a greater or lesser risk of progression of this pain.

Capsaicin was also found to stop pain sensation via multiple molecular mechanisms. It inhibits specific voltage-gated sodium channels (VGSC) in the trigeminal ganglion neuron and prefrontal cortex pyramidal neurons, thus not allowing the production of an action potential in the nerves of the pain pathway, stopping any pain perception from occurring [8,21]. It also releases cytochrome C in glial cells causing apoptosis [52]. This double action of capsaicin on exciting neurons due to its pro-inflammatory action, through apoptosis, and its VGSC inhibition. At this stage, it proves ambiguous as to whether capsaicin can treat chronic neuropathies through CNS modulation as it depends on which of its effects (whether it is CNS hypersensitisation or inhibition of pain sensation) are more profound. 

An interesting exploratory aspect of capsaicin is its activity and bioavailability in the CNS. Attributes of a high bioavailability and absorption in the CNS means it may reach up to 94% absorption [1,53]. Topical capsaicin absorbs well in skin due to its lipophilicity, which means it can pass through the stratum corneum layer of the skin and easily enter the systemic circulation. More capsaicin in the circulation means more received by the body, but some areas, like the CNS and the skin, have slower capsaicin metabolism even with a high bioavailability, suggesting a lengthened time for it to act on the TRPV1 receptors on the spinal cord and the sensory ganglia. However, there are other factors affecting the absorption of capsaicin. These include the dose of application and its tendency to have a greater effect locally [54]. Regarding the dose, a much higher dose would be required to have any recordable impact on the CNS, but the initial burning sensation post-application would be expected to increase with the dose of capsaicin administered. Therefore, a particularly useful next step would be studying the topical effects of capsaicin on the CNS, particularly the sensory ganglia, with different doses of topical capsaicin with stepwise dose application of capsaicin, and the effect when systemic capsaicin is used directly as such research does not yet exist in any substantive form. 

Overall, it has been widely accepted that TRPV1 channels are defunctionalised by capsaicin, and that their presence throughout the CNS makes it highly likely that capsaicin affects the CNS too. The specific mechanisms underlying this, however, are not certain, and need to be explored further in order to ascertain why this is the correct method of action. The magnitude of the effect on the CNS is uncertain due to factors like dose applied, capsaicin administration modality, absorption levels, and presence in the CNS not being fully understood. To date, it has not been compared in any meaningful trial data that explicitly test the means of these.

Within the clinical setting, capsaicin shows promise for the advancement of treatment methods providing a greater level of pain relief and improving prognosis compared to the pre-existing treatment methods. In studies exploring the use of capsaicin in chronic post-surgical pain, it was found that when using a high concentration of intradermal capsaicin within a rat test-population, it provided a reduced sensitivity to pain and temperature in the test population compared to the control study, showing that capsaicin has beneficial characteristics as an analgesic by reducing pain and temperature nociception, suggesting a possible effect on the spinal cord tracts responsible (namely the lateral spinothalamic tract) [31]. When applying a similar hypothesis to human studies and systematic reviews, it was found that when applied topically at high levels, capsaicin was able to expeditiously alter sensitivity, producing a greater tolerance to neuropathic pain in the peripheral nervous system [33]. The expenditure of these patches is greater than the standardised treatment; however, with the additional benefit of a single application reducing issues with compliance, capsaicin could be considered more cost effective and advantageous in producing and maintaining more desirable outcomes. Capsaicin is also shown to be of benefit to those who are afflicted by cluster headaches. Studies show it mediates its effects through the attenuation of substance P at the nerve terminals, altering their sensitivity (40). It also impacts the trigeminal nociceptive neurons and their expression of TRPV1 ion channels. When fully depolarised, nociceptive areas become desensitised; these consequentially correct the chemical dysregulation and provide relief to the experienced symptoms more instantaneously than seen in pre-existing treatments [38].

Furthermore, in phantom limb pain although pathophysiology was previously thought of as confined to the periphery, studies have shown that the central nervous system may play a key role within this pain mechanism owing to somatosensory cortex remodelling. Investigations have demonstrated that patients who applied capsaicin to their amputation site found a reduction in both experiences of phantom limb pain and in site pain for the following four weeks without the presence of side effects [49]. Also, in patients with phantom limb pain, research in neuroimaging using functional MRI suggests that they experience cortical remapping, so that sensory modality is reallocated, restoring the mapping of the brain. It was found that those who experience relief from the symptoms of phantom limb pain due to the use of capsaicin also experienced a greater degree of cortical remapping, thus demonstrating that the direct influence of peripheral pathways causing PLP can be resolved by the application of capsaicin and that there is a link between capsaicin’s effects in the peripheral and central nervous systems implicated in this [46]. 

The application of all this data is that capsaicin could be a possible treatment for chronic neuropathic pains like cluster headache, phantom limb pain, and post-surgical chronic pain with the possibility of better outcomes and quicker relief, unlike other analgesics, which have more side effects and drug-to-drug interactions. However, this must be first confirmed by proving capsaicin’s efficacy in isolation, but also in comparison to the established and existing treatments.

## 4. Limitations

Our study analysed 58 papers of different kinds, selected based on relevance to our study. While our literature search was thorough, one limitation is the small sample size, and large variety in the types of papers we included, being a mix of systematic reviews, journal articles, narrative reviews, clinical trials, and in vivo and in vitro studies. This makes the comparison of evidence more problematic and not possible directly. While our study primarily investigated topical capsaicin, the papers we analysed on capsaicin looked at a variety of methods of administration, including injection directly into the periaqueductal grey, to gain more insight into how safely and effectively capsaicin interacts with the CNS. This limits the number of changes we can firmly say were caused by topical administration, though this did give valuable insight into specific molecular mechanisms with direct exposure. While many studies point towards topical capsaicin modulating the CNS, a mechanism for this change is currently speculative and is an important avenue to explore in future in vivo studies.

## 5. Conclusions

Capsaicin, a substance present in red chilli, can stimulate TRPV1. This activation makes the specific area more sensitive to heat, acidic conditions, and natural substances that promote a physiological response. Capsaicin is found to be an effective analgesic with a rapid onset and long-lasting effect.

Limited research has been conducted on the potential influence of certain structural and functional alterations in the central nervous system on the outcomes of chronic neuropathic pain. Also, the evidence of whether topical capsaicin may be involved in regulating these changes is unclear. Hence, more research is necessary to gain a full understanding of this complex mechanism. Methods of research that may improve the range of knowledge in this field may include wider observational studies in populations already taking capsaicin, such as a cohort study with follow up over a specified time period. Controlled trials may also prove beneficial, whereby patients are investigated for any changes in the CNS both in a control group and a group treated with topical capsaicin, observing for any modulation, and if present any difference in CNS modulation.

The findings from this study indicate that not only does neuropathic pain cause changes in the structure and function of the peripheral nervous system (PNS), but it also affects the central nervous system (CNS), particularly in the somatosensory cortex. The significance of capsaicin is supported by the different pharmaceutical products and medical uses attributed to it, like the capsaicin 8% patch that is used for treating neuropathic pain.

As a result, there are promising pharmacological approaches that we can expect to see in the coming years. These include the development of new pharmaceutical formulations, the creation of new analogues, or focusing on the receptor TRPV1, which is activated by capsaicin. However, more studies such as those above, alongside the use of a quantitative measure, such fMRI, in patients with neuropathic pain should be conducted to allow a more comprehensive approach in understanding capsaicin’s mechanism of action.

## Data Availability

All data generated or analysed during this study are included in this article and no additional source data are required.
